# Chronic Mild Stress (CMS) in Mice: Of Anhedonia, ‘Anomalous Anxiolysis’ and Activity

**DOI:** 10.1371/journal.pone.0004326

**Published:** 2009-01-29

**Authors:** Martin C. Schweizer, Markus S. H. Henniger, Inge Sillaber

**Affiliations:** 1 Affectis Pharmaceuticals AG, Martinsried, Germany; 2 Max-Planck-Institute of Psychiatry, Munich, Germany; James Cook University, Australia

## Abstract

**Background:**

In a substantial proportion of depressed patients, stressful life events play a role in triggering the evolution of the illness. Exposure to stress has effects on different levels in laboratory animals as well and for the rat it has been shown that chronic mild stress (CMS) can cause antidepressant-reversible depressive-like effects. The adoption of the model to the mouse seems to be problematic, depending on the strain used and behavioural endpoint defined. Our aim was to evaluate the applicability of CMS to mice in order to induce behavioural alterations suggested to reflect depression-like symptoms.

**Methodology/Principal Findings:**

A weekly CMS protocol was applied to male mice of different mouse strains (D2Ola, BL/6J and BL/6N) and its impact on stress-sensitive behavioural measures (anhedonia-, anxiety- and depression-related parameters) and body weight was assessed. Overnight illumination as commonly used stressor in CMS protocols was particularly investigated in terms of its effect on general activity and subsequently derived saccharin intake. CMS application yielded strain-dependent behavioural and physiological responses including ‘paradox’ anxiolytic-like effects. Overnight illumination was found to be sufficient to mimic anhedonic-like behaviour in BL/6J mice when being applied as sole stressor.

**Conclusions/Significance:**

The CMS procedure induced some behavioural changes that are compatible with the common expectations, i.e. ‘anhedonic’ behaviour, but in parallel behavioural alterations were observed which would be described as ‘anomalous’ (e.g. decreased anxiety). The results suggest that a shift in the pattern of circadian activity has a particular high impact on the anhedonic profile. Changes in activity in response to novelty seem to drive the ‘anomalous’ behavioural alterations as well.

## Introduction

Animal models are important research tools in psychiatry and should mimic some of the human conditions of interest. According to DSM-IV [1], major depression is characterised by either depressed mood or anhedonia, in combination with four additional symptoms related to weight changes, sleep disturbances, psychomotor agitation or retardation, fatigue, feelings of worthlessness or guilt, diminished cognitive functioning, or recurrent thoughts of death. The presence of some of these symptoms can be defined operationally (e.g., loss of appetite and weight, sleep disturbances, cognitive and psychomotor changes), and thus can be assessed in laboratory animals.

Stressful experiences have been reported to favour the development of depression in humans [e.g. 2,3]. Therefore, in order to provoke depressive-like behavioural changes, some animal models for this phenotype are generated by exposing them to stressful situations [4]–[8]. In rats, application of chronic mild stress (CMS) procedures resulted in a variety of behavioural, neurochemical, neuroendocrine and neuroimmune alterations resembling some of the dysfunctions observed in human depression [9]–[16]. Therefore, the CMS model, developed by Willner and colleagues [4], has attracted a lot of interest due to its potential of combining several validity criteria requested for an animal model of depression [16], [17]. In terms of symptoms evoked by CMS, the induction of anhedonia was the primary focus in this model [4]. Anhedonia, a core symptom of depression, was modelled by inducing a decrease in responsiveness to rewards reflected by a reduced consumption and/or preference of sweetened solutions. In a more recent review, Willner [17] summarises results of positive reproduction of CMS-induced anhedonia as well as ‘anomalous’ findings. In this context the attributes ‘anomalous’ or ‘paradox’ refer to findings that include CMS-induced anxiolysis and hyperlocomotion and apparently are contrary to ‘classic’ comorbidities of depression-related behaviour such as increased anxiety and reduced locomotor activity [17], [18]. Most studies were performed in rats but the few that used mice generally point towards the applicability of a non-standardised CMS procedure to induce anhedonic behaviour in mice as well. One reason for the adoption of this approach to mice was the introduction of genetic mouse models as tools in psychiatric disorder research. Using mice with specific genetic modifications in suspected vulnerability genes in combination with CMS as environmental factor will allow scrutinising the role of both, the candidate gene and stress in a genetically predisposed animal, as risk factors for depression.

Studies published on the effects of CMS on mouse behaviour agreed on the importance of the strain used and showed that some strains are responsive to the CMS procedure, a finding which strongly depends on the respective endpoint defined [19]–[24]. The co-occurrence of CMS-induced alterations in palatable liquid consumption and other behavioural and physiological parameters, suggested to reflect depressive-like behaviour, has rarely been studied in mice. Pothion and colleagues [21] investigated three different mouse strains in terms of sucrose consumption, body weight changes, coat state, spontaneous alternations in the Y-maze and spatial learning in the Morris water maze test. Only in one strain (CBA/H) a decrease in sucrose consumption was found along with changes in physiological or behavioural parameters. Therefore, a CMS-induced reduction of sucrose consumption was not predictive of the occurrence of any other parameter assessed in the study. Applying a different chronic stress procedure in BL/6N mice, comprising more severe stressors presented in a more sequential than unpredictable manner, Strekalova and colleagues [25] described an association of a stress-provoked decrease in sucrose preference and behavioural measures that were restricted to increased passivity in the forced swim test (FST) as well as decreased novel object exploration. An increase in anxiety-related behaviour and locomotor disturbances were found to be induced by the stress procedure independent of the induction of “anhedonic” behaviour [25]. In other studies using the CMS procedure, an increase in immobility in the FST [26]–[28], decreased grooming [23], altered performance in cognitive tasks [29] as well as changes in anxiety-like behaviour [20], [24] were observed. These behavioural measures were considered to reflect additional depression-related symptoms [17]. Unfortunately consumption of sweetened solutions was not assessed in all these studies.

A CMS model with a decrease in intake and preference of sweetened solutions as central readout presents specific advantages. Contrary to operant behaviour it can be implemented without cost-intensive apparatus, it is non-invasive (in contrast to e.g. intracranial self-stimulation), and the progressive evolution of a depression-related symptom as well as the time course of antidepressant-induced resolution of the symptom can be traced by repeated measurements. The latter is rather difficult for a variety of test paradigms used to assess, for example, anxiety-like behaviour. As stated by Anisman and Matheson [5], an appropriate model of depression requires that multiple behavioural tests be employed to approximate the range of symptoms that characterise depressive illness. Therefore, the aim of our studies was to further investigate the feasibility to establish a CMS model which includes the measurement of intake and preference of a sweetened liquid and addresses additional indicators of depressive-like behaviour. We used a stress-procedure which included most of the commonly used “mild” stressors [30] but was devoid of food or water deprivation. Measurements were performed 2–3 times per week with the aim to possibly gain information on a direct effect of the precedent stressor on “hedonic” behaviour. CMS effects on intake of a 0.2% saccharin solution and body weight gain were assessed in different mouse strains as the vulnerability for stress-induced changes is supposed to be genetically determined. In selected mouse strains, we investigated the influence of CMS on anxiety-related behaviour and on passive behaviour in the FST. The fluctuations in saccharin intake observed in the first experiments showed a tendency of association to the precedent stressor which according to our interpretation had some impact on nocturnal activity of the mice. Therefore, an independent experiment aimed at detecting the influence of overnight illumination as particular stressor on subsequent saccharin intake and activity in parallel.

## Materials and Methods

### Animals and housing conditions

Male mice from seven strains (Balb/cOla, C57BL/6JOla, C57BL/6N, DBA/2Ola, DBA/2JIco, FVB/N, NMRI) - referred to as Balb/c, BL/6J, BL/6N, D2Ola, D2JIco, FVB and NMRI respectively - were purchased from Harlan-Winkelmann GmbH (Borchen, Germany; Balb/c, BL/6J, D2Ola, FVB and NMRI) and Charles River GmbH (Sulzfeld, Germany; BL/6N and D2JIco). Upon arrival, the animals were singly housed in Macrolon Type II cages under standard laboratory conditions (temperature 21±1°C, rel. humidity 40–60%, 12h∶12h light/dark cycle, lights on 6 A.M.), had free access to food and water and were allowed to habituate to the novel environment for at least 2 weeks. Afterwards, the basal consummatory behaviour for water and either saccharin or sucrose (intake and preference) was determined. Based on these parameters and on body weight, mice were matched and assigned to stress and control groups. Animal experiments were performed in accordance with the NIH Guide for the Care and Use of Mammals in Neuroscience and Behavioural Research and the Guide for the Care and Use of Laboratory Animals of the Government of Bavaria, Germany.

### CMS Paradigm

The CMS procedure followed a fixed weekly schedule of commonly used mild stressors such as repeated cold stress (4°C), space reduction in the homecage, changed cages within CMS group, cage tilt, empty cage, intermittent air puff, wet bedding, white noise, overnight illumination and social interaction with other animals of the CMS group. The particular context (stressor applied during preceding dark phase) of the 2 hrs liquid consumption measurement intervals involved overnight illumination (Sunday–Monday), wet cage and cage tilt (Tuesday–Wednesday and Thursday–Friday, long-term CMS in BL6/J and D2Ola mice) and changed cages (Wednesday–Thursday, shorter CMS in BL6/J, BL6/N and the other strains), respectively. For details see Table 1.

**Table 1:** Weekly CMS schedule

	LIGHT PHASE		DARK PHASE	
	First half	Second half	First 2 hrs	Remaining 10 hrs
**Mon**	Repeated Cold Stress (2×30 min)	Cold Stress (30 min)	***2 hrs liquid intake measurement*** *	Homecage Space Reduction
**Tue**	Changed Room	Air Puff (3×3 intermittent)	Wet Cage	Wet Cage
**Wed**	Wet Cage	Social Interaction	Foreign Cage	Foreign Cage
**Thu**	Foreign Cage	Social Interaction	***2 hrs liquid intake measurement*** *	Cage Tilt
**Fri**	Empty Cage	Changed Room	White Noise&Strobe	White Noise&Strobe
**Sat**	White Noise	Pause/Changed Room	Pause/Changed Room	Pause/Changed Room
**Sun**	Pause/Changed Room	Pause/Changed Room	Overnight Illumination	Overnight Illumination

* Exemplified for short-term CMS experiments. During long-term CMS liquid intake was assessed on Mon, Wed and Fri in the same interval.

### Behavioural Tests

#### Consummatory Behaviour

Extensive preliminary tests for preference of cage side, saccharin preference over water, saccharin concentration and one- versus two-bottle paradigm conducted before the CMS period in the long-term CMS paradigm resulted in the following measurement protocol. Liquid intake was determined 3 times a week (Monday, Wednesday, Friday) in a two-bottle paradigm by weighing the bottles before and after the first 2 hours of the dark phase ( = measurement interval of liquid consumption) on the basis of D'Aquila's studies [31]. Sweet solutions were offered on the preferred right cage side, as a shifting from the preferred solution at the preferred cage side to water at the other side might more closely model anhedonic behaviour. With the exception of the experiment in BL/6N mice, a 0.2% saccharin solution was presented as palatable liquid. Using BL/6N mice and applying a different stress procedure Strekalova et al. [25] reported a decrease in sucrose preference coupled with other behavioural alterations. In order to test whether our CMS procedure induces a profile in BL/6N mice comparable to the reported one we stayed with the presentation of sucrose and changed the stress protocol only.

In the other experiments saccharin was chosen to avoid a caloric impact of the sweetened liquid consumption on the CMS effects. Since saccharin presentation 3 times a week might have worn down the hedonic value of saccharin also in control mice, in the shorter experiments sweet solutions were presented only twice a week on Monday and Thursday.

#### Dark/Light Box (DaLi)

Anxiety-related behaviour was assessed in a DaLi (dark compartment 15×20×25 cm, light compartment 30×20×25 cm, connected by a 4 cm long tunnel). The light compartment was illuminated with 700 lx (for D2Ola and BL/6J mice) and 50 lx (for BL/6N animals) cold light, whereas in the dark compartment the illumination level was 5 lx. Animals were placed in the dark compartment and the time spent in, the latency to first entry (all four paws) and the number of entries into each compartment was recorded for 5 min using the ANY-maze software (Stoelting Co., Wood Dale, IL).

#### Forced Swim Test (FST)

Each animal was placed into a beaker (diameter 12 cm, height 24 cm) filled with water (temperature 25−26°C) to a height of 12 cm for a test period of 5 min. The parameters floating (immobility with only small movements to keep balance), swimming and struggling (vigorous attempts to escape) were scored throughout the 5 min test period by a trained observer blind to the treatment and recorded using the ANY-maze software.

#### Modified Hole Board Test (mHb)

The mHb test (for details see [32]) was performed at 6 P.M. at 60 lx (long-term CMS) and 30 lx (BL/6N) respectively. The apparatus consisted of a dark grey PVC box (100×50 cm, 50 cm height) containing a board (70×20×0.5 cm) in the centre which was equipped with 12 cylinders (“holes”, 2 cm height×3 cm diameter). Behavioural parameters such as time spent on board (with all four paws), rearing (standing in an upright position on the hindpaws) and locomotor activity (total distance travelled) were recorded during a 5 min session using the ANY-maze software.

### Experimental Design

#### Assessment of CMS effects on consummatory behaviour

a) Long-term CMS in BL/6J and D2Ola mice: Animals (n = 24 per strain) underwent preliminary tests to develop the liquid intake measurement protocol. Subsequently, at an age of 36 weeks, the CMS regimen was applied for 13 weeks (for each strain control and stress group n = 12). During the CMS period the saccharin consumption was measured 3 times a week (Monday, Wednesday and Friday) for 10 weeks.

b) Short-term CMS in other mouse strains: An independent batch of BL/6J animals (aged 12 weeks at the beginning of CMS application, n = 10/group) was tested to assess reproducibility of CMS-effects on consummatory behaviour. BL/6N mice (aged 17 weeks at the beginning of the stress period, 9 control and 20 CMS animals) were offered a 2% sucrose solution for 4 weeks of CMS.

Consummatory behaviour was accordingly assessed also in additional mouse strains during 3–4 weeks of CMS. The animals were aged 12 weeks (Balb/c, n = 10/group), 7 weeks (D2JIco, n = 24/group) and 21 weeks (FVB and NMRI, n = 8/group), respectively, at the beginning of the stress period.

#### Assessment of CMS effects on anxiety- and depression-related behaviour

a) Long-term CMS in BL/6J and D2Ola mice: The long-term (13 weeks) stressed BL/6J and D2Ola mice were tested in the DaLi after 11 weeks and the FST after 12 weeks of CMS exposure. The testing times were 6 P.M. and 8 A.M., respectively. The week after, mice were tested in the mHb (6 P.M., 60 lx).

b) Short-term CMS in BL/6J and BL/6N mice: The short-term (4 weeks) stressed BL/6J mice were tested for anxiety-related behaviour in the DaLi after 2 weeks and for depression-related behaviour in the FST after 4 weeks of CMS. The FST was conducted from 9 A.M. to 12 P.M. under standard laboratory conditions. Before being tested for CMS effects on sucrose consumption as described above, the BL/6N mice were characterised in their basal ( = non-stressed) behaviour in several paradigms (DaLi, elevated plus maze, open field, resident-intruder test), then following the CMS procedure for 4 weeks were re-tested in the above mentioned paradigms (plus FST) and subjected to additional 3 weeks of CMS. Stress effects on behaviour were finally assessed in the mHb. All behavioural tests were conducted during the first 2 hours of the dark phase (corresponding to the consumption measurement interval) either at reduced illumination (mHb: 30 lx) or under red light conditions (all other tests).

#### Effects of overnight illumination as sole stressor in BL/6J mice

For 4 weeks, BL/6J mice (40 stressed and 36 control animals aged 7 weeks at the beginning of the stress period) were exposed to overnight illumination (light stress, LS) twice a week (Sunday and Wednesday) and the saccharin consumption and homecage activity was measured during the following dark period (Monday/Tuesday and Thursday/Friday, respectively). No further stressors were applied. To assess their general activity pattern, mice were monitored in their homecages in side-view using small CCD cameras and the ANY-maze software. Parameters such as locomotor activity, rearing and climbing behaviour were determined. Data were analysed for the 2 hrs consumption measurement interval as used in the CMS experiments and for the subsequent 22 hours to yield a 24 hrs consumption/activity profile.

### Statistical Analysis

Data were analysed using STATISTICA Software 6.0 (Statsoft Inc., Tulsa, US). A two-sided Student's t-test was applied for comparison of two experimental groups. For time series analysis (consummatory behaviour and locomotor activity) repeated measures ANOVA with Greenhouse-Geisser correction for non-sphericity was used. For traceability reasons uncorrected degrees of freedom plus the respective correction factor ε and corrected F values are shown. When repeated measures ANOVA revealed a significant interaction effect of the factors time and group, data were further analysed with pairwise comparisons for each time point (Student's t-test). The inter-strain comparison of saccharin intake (see supplementary material) was analysed using one-way ANOVA followed by Tukey post-hoc testing. For the analysis of body weight gain averages were calculated for the CMS period and compared using a two-sided Student's t-test. Effects were considered significant when p<0.05.

## Results

### CMS effects on consummatory behaviour in different mouse strains

**Figure 1:**
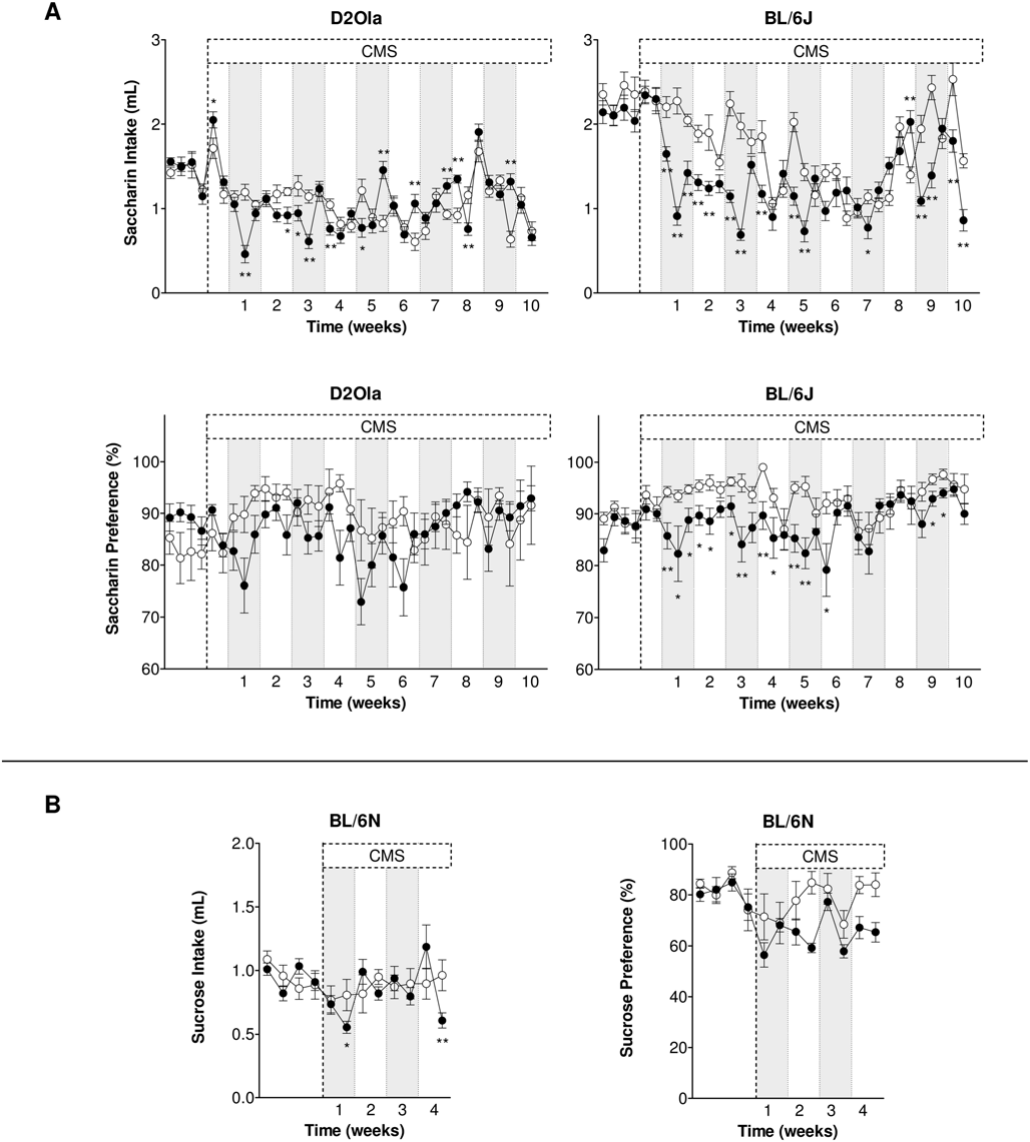
CMS effects on palatable liquid consumption per 2 hrs in D2Ola, BL/6J and BL/6N mice. (A) Effects of CMS on saccharin intake and preference over a period of 10 weeks in D2Ola and BL/6J mice (measurement 3x/week). (B) CMS effects on sucrose intake and preference over a period of 4 weeks in BL/6N mice (measurement 2x/week). Saccharin concentration: 0.2%, sucrose concentration 2%. White circles: control group, black circles: CMS group. First 4 data points of each graph represent basal consumption. Data represent mean±SEM, n = 9–20/group. * p<0.05, ** p<0.01 pairwise between-group comparisons (Student's t-test).

For saccharin intake of long-term CMS exposed D2Ola mice repeated measurement ANOVA yielded no significant effect for the factor “group” ( = CMS). After beginning of the CMS period, at the times marked in Figure 1A, differences in saccharin consumption in the experimental groups could be detected (interaction group×time: F_31,682_ = 9.29; p<0.01; ε = 0.359), albeit stating in some measurements an elevated intake in CMS mice compared to the control group. CMS had no effect (factor group) on the preference for saccharin in D2Ola mice (Figure 1A), no significant interaction resulted from the factors group and time.

In BL/6J mice, statistical analysis revealed a reduced saccharin intake (factor group: F_1,22_ = 12.30; p<0.01). This difference also appeared in the interaction of the factors group×time (F_30,660_ = 14.27; p<0.01; ε = 0.366), whose details are illustrated in Figure 1A. CMS led to a decreased saccharin preference (factor group: F_1,22_ = 4.92; p<0.05; interaction group×time F_30,660_ = 2.57; p<0.01; ε = 0.298) of BL/6J animals in the long-term CMS paradigm (see Figure 1A). After four weeks CMS exposure, these findings could be replicated for an independent batch of BL6/J mice (data not shown) regarding their saccharin intake (factor group: F_1,18_ = 24.98; p<0.01; interaction group×time: F_5,90_ = 4.33; p<0.01; ε = 0.584) but not in respect of their saccharin preference.

Further, as illustrated in Figure S1, the CMS regimen affected saccharin intake of Balb/c mice (factor group: F_1,18_ = 6.03; p<0.05; interaction group×time: n.s.), FVB animals (factor group: F_1,14_ = 14.09; p<0.01; interaction group×time: F_8,112_ = 5.63; p<0.01; ε = 0.536), NMRI mice (factor group: F_1,14_ = 6.75; p<0.05; interaction group×time: n.s.) and D2JIco mice (factor group: F_1,45_ = 12.90; p<0.01; interaction group×time: n.s.). Inter-strain comparison for basal saccharin consumption and percental change induced by CMS is provided in Table S1.

In BL/6N mice, repeated measurements ANOVA yielded no CMS effect on sucrose intake in terms of factor group but an interaction effect of factors group×time (F_7,245_ = 5.89; p<0.01; ε = 0.618). Sucrose preference was significantly decreased by CMS (factor group: F_1,35_ = 13.48; p<0.01; interaction factors group×time: F_7,245_ = 3.32; p<0.01; ε = 0.629). The sucrose consumption of BL/6N mice is illustrated in Figure 1B.

### CMS effects on anxiety- and depression-related behaviour and body weight gain

BL/6J mice were selected to be further investigated as this mouse strain is commonly involved in the generation of transgenic mice. We were interested in the D2Ola mice as we observed in previous studies that D2Ola compared to BL/6J responded with a reduced inhibitory HPA axis feedback to a single stress exposure [33]. BL/6N mice were integrated in our studies as Strekalova et al. [25] showed an association of anhedonic and depressive-like behaviour for a subgroup of this mouse strain after chronic stress.

#### Dark/Light Box (DaLi)

**Figure 2:**
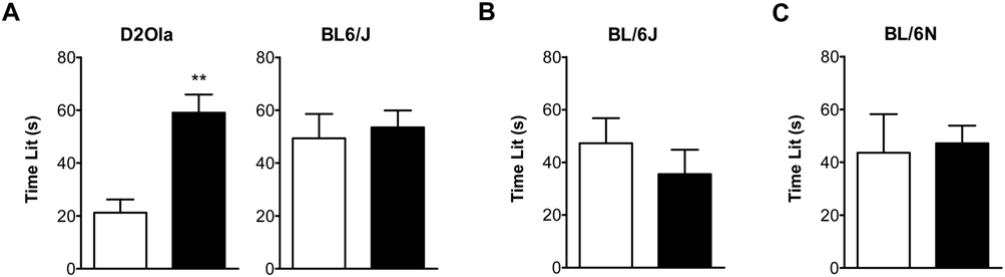
CMS effects on time spent in the lit compartment of the DaLi. (A) In D2Ola mice long-lasting CMS exposure (11 weeks) led to behavioural changes reflected by an increase in time spent in the lit compartment (700 lx) compared to the control group. In BL/6J mice with similar CMS experience no changes were observed. (B) BL/6J mice with CMS experience of 2 weeks were also unaffected in terms of time spent in the lit compartment (700 lx). (C) BL/6N mice with CMS experience of 4 weeks did not differ from control mice in the parameter time lit, when tested at a moderate illumination level of 50 lx in the lit compartment. Data represent mean + SEM, n = 9–20/group. White bars: control group, black bars: CMS group. ** p<0.01 (Student's t-test).

Long-term CMS application caused D2Ola mice to spend significantly more time in the lit compartment compared to controls (t_21_ = 4.55; p<0.01), whereas stressed BL/6J mice of the same experiment did not show an altered behaviour in the DaLi paradigm (Figure 2A). The latter finding was in line with results obtained using the same strain in the DaLi test after 2 weeks of CMS (Figure 2B). In BL/6N mice, the time spent in the lit compartment was not affected by 4 weeks of CMS pre-experience (Figure 2C).

#### Modified Hole Board (mHb)

**Figure 3:**
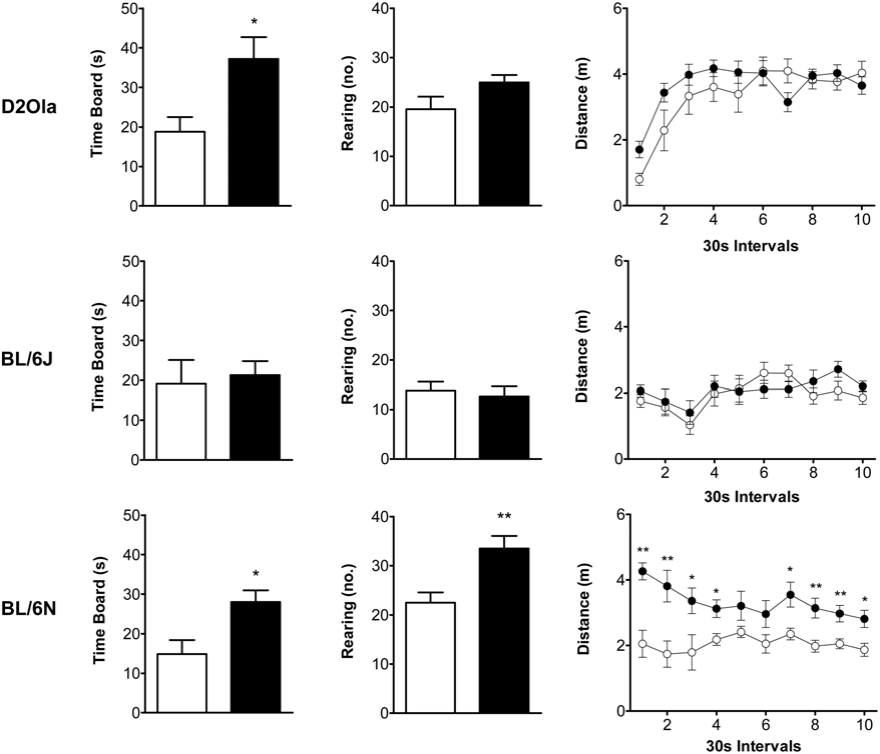
CMS effects on behaviour in the mHb. In D2Ola mice 13 weeks of CMS led to an increase in time spent on the board, whereas BL/6J animals of the same experiment did not show altered behaviour (time board, number rearings and distance travelled). Mice of the BL/6N strain (7 weeks of CMS experience) showed significant increases in the parameters time board, number of rearings and an increased locomotor activity. Illumination conditions: 60 lx (D2Ola, BL/6J) and 30 lx (BL/6N), respectively. Data represent mean±SEM, n = 9–12/group. White circles and bars: control group, black circles and bars: CMS group. * p<0.05, ** p<0.01 (Student's t-test).

Behaviour of the different strains in the mHb is displayed in Figure 3. Long-term stressed D2Ola animals spent more time on the board (t_20_ = 2.78; p<0.05), while rearing behaviour and locomotor activity tended to result in a slight increase due to CMS. BL/6J animals did not show any behavioural changes induced by CMS in the mHb test. However, mHb testing revealed strong effects of 7 weeks of CMS on behaviour of BL6/N mice: Time spent on board, number of rearings and locomotor activity were significantly increased in stressed animals (time board: t_16_ = 2.91; p<0.05, rearing: t_16_ = 3.23; p<0.01, locomotion: factor group F_1,16_ = 14.82; p<0.01; interaction group×time: F_9,144_ = 1.97; p<0.05).

##### Forced Swim Test (FST)

**Figure 4:**
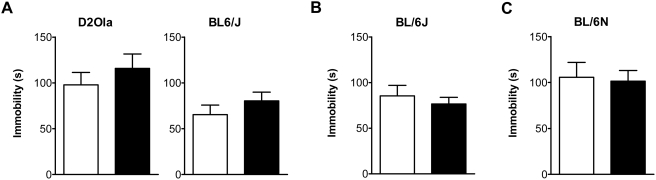
CMS effects on immobility in the FST. (A) 12 weeks of exposure to CMS had no effect on immobility time of D2Ola and BL/6J mice in the FST. (B) 4 weeks of CMS exposure had no effect on immobility time of BL/6J mice. (C) After 4 weeks of CMS exposure, BL/6N mice were subjected to the FST at the end of week 5. No changes in immobility time compared to the control group could be observed. Time of test: 8 A.M. (A,B) and 6 P.M. (C), respectively. Data represent mean + SEM, n = 9–20/group. White bars: control group, black bars: CMS group.

In none of the investigated mouse strains an increased immobility in the FST due to CMS could be observed (p>0.05) (Figure 4).

#### Body weight gain

**Figure 5:**
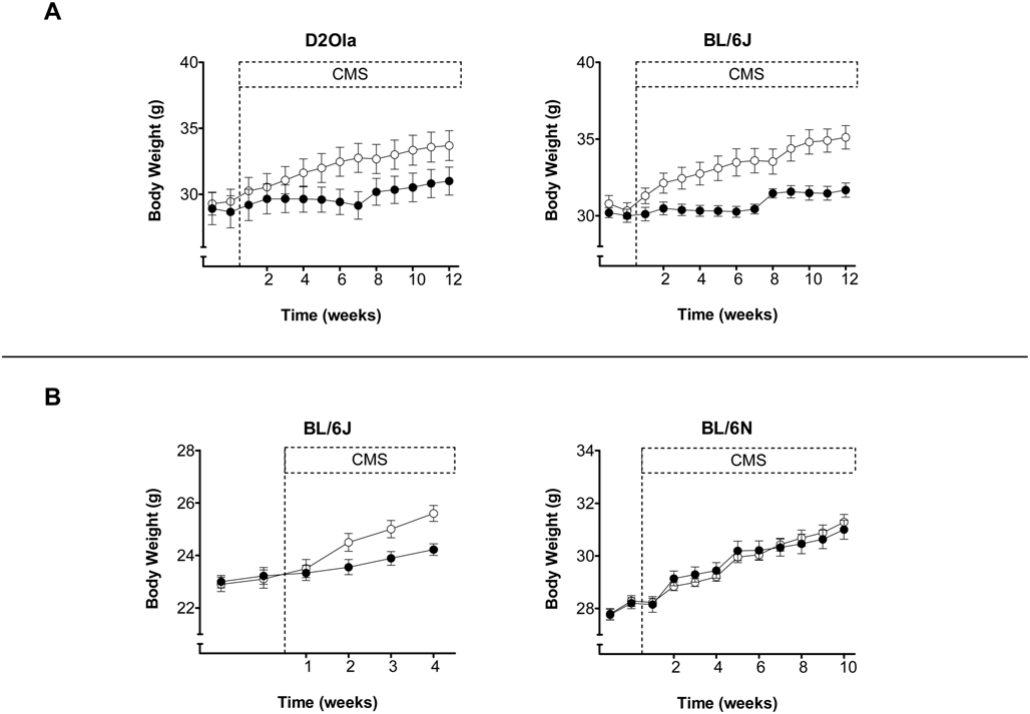
CMS effects on body weight. (A) The illustrated time courses of body weights are characterised by a flattened mean slope in CMS animals compared to controls, thus revealing a reduced body weight gain due to CMS in both D2Ola and BL/6J mice of the long-term CMS experiment. (B) In the shorter CMS experiments a similar reduction of body weight gain due to CMS could be observed in younger BL/6J but not in BL/6N animals. Data represent mean±SEM, n = 9–20/group. White circles: control group, black circles: CMS group.

As illustrated in Figure 5A, CMS reduced body weight gain in D2Ola and BL/6J mice during the 13 weeks CMS schedule (t_21_ = 2.22; p<0.05 and t_22_ = 4.00; p<0.01, respectively). This result could be confirmed for mice of the latter strain in the shorter CMS experiment (t_17_ = 2.93; p<0.01), whereas CMS had no effect on body weight gain in BL/6N animals (Figure 5B).

### Effects of overnight illumination as sole stressor in BL/6J mice

#### Consummatory behaviour

**Figure 6:**
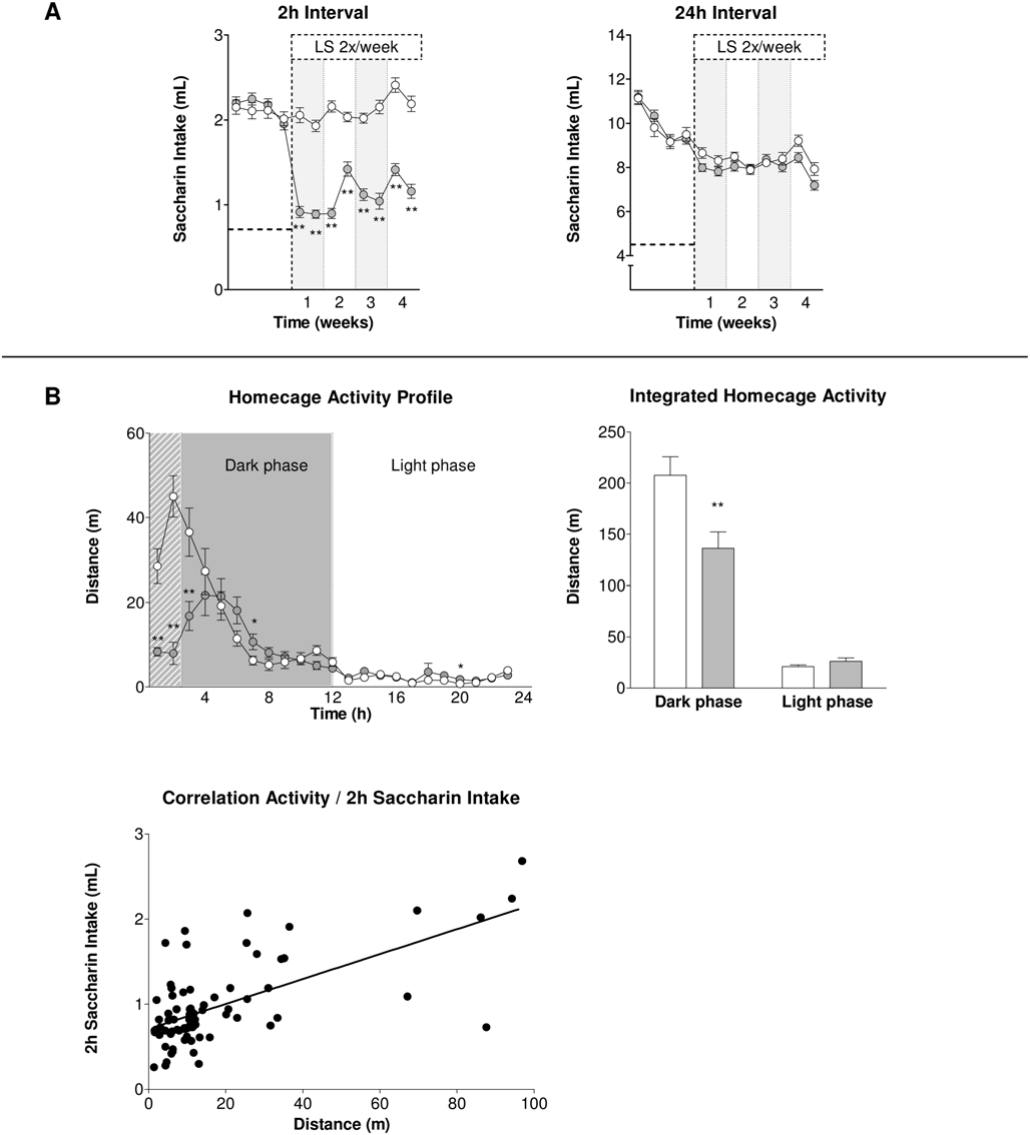
Effects of Light Stress (LS) on subsequent saccharin intake and homecage activity in BL/6J mice. (A) LS resulted in a reduction of saccharin intake during the first 2 hrs interval while there was no effect on overall 24 hrs saccharin intake after LS. The dashed horizontal lines at y = 0,72 mL and y = 4,80 mL, respectively, represent basal water intake when only water is available in a two-bottle paradigm during the corresponding measurement interval. (B) Profiling of homecage activity for a total of 24 hrs (t_0_ = beginning of the dark phase) revealed a distinct decrease during the first hours of the dark phase (which comprised the 2 hrs saccharin measurement interval, hatched area) that was positively correlated with the respective 2 hrs saccharin intake (Pearson's r = 0.64, p<0.01, n = 76, i.e. 40 LS and 36 control animals). Data represent mean±SEM, n = 36–40/group. White circles and bars: control group, grey circles and bars: light stress group. * p<0.05, ** p<0.01 pairwise between-group comparisons (Student's t-test).

Overnight illumination as sole stressor was able to considerably decrease saccharin intake of BL/6J animals during the 2 hrs measurement interval (factor group: F_1,74_ = 185.96; p<0.01; interaction group×time: F_7,518_ = 6.22; p<0.01; ε = 0.715), an effect that almost retained significance throughout the 24 hrs measurement interval (factor group: F_1,74_ = 3.60; p = 0.06; interaction group×time: n.s.), for details see Figure 6A.

#### Homecage activity

BL/6J animals exposed to overnight illumination as sole stressor showed reduced homecage activity in the 24 hrs interval (factor group: F_1,62_ = 6.73; p<0.05), whereupon localisation of differences revealed a distinctly decreased activity during the first 3 hours of the dark phase (interaction group×time: F_22,1364_ = 12.25; p<0.01; ε = 0.149), i.e. comprising the time span during which both 2 hrs saccharin measurement took place and control animals peaked in homecage activity (for details see Figure 6B). Overnight illumination delayed (and decreased) peak activity in the presence of the saccharin solution in LS animals by around 2–3 hours. Two-way ANOVA of homecage activity during dark and light phase yielded an interaction effect of light stress and daytime (F_1,62_ = 10.86; p<0.01), whose details are shown in Figure 6B. Here, overnight illumination could be shown to reduce homecage activity during the dark phase (p<0.01) while light phase activity remained unchanged. Correlation of homecage activity of both control and CMS animals during the 2 hrs consumption measurement interval with 2 hrs saccharin intake revealed Pearson's r = 0.64 (p<0.01), see Figure 6B.

## Discussion

The data presented in this study were derived during the development of a CMS protocol focussed on consumption of sweet solutions as central readout measure for hedonic/motivational behaviour in mice. We used a stress-procedure which included most of the commonly used “mild” stressors [16], [17], [30] but was devoid of food or water deprivation. Five out of seven mouse strains investigated responded with a decrease in the consumption of the sweetened solution compared to the respective control group. As summarised by Willner [17], a variety of chronic stress protocols has been able to yield an anhedonic phenotype in rodents regardless of particular content or timetabling of microstressors in the respective CMS schedule. Strain-dependent effects of CMS on consumption of palatable solutions of mice have been reported earlier [20], [21], [24].

These general findings could be confirmed in our study for stressed BL/6J, D2Ola, D2JIco, FVB and BL/6N mice despite differences in the designs of the experiments. However, the effects of CMS appeared not to be enduring, since the saccharin intake of the respective control group decreased to the level of stressed animals after 4–6 weeks in the long-term CMS experiment. Trying to re-establish their working CMS protocol after moving to a new laboratory, Willner and colleagues reported similar problems of an initially obtained anhedonic CMS effect in rats that vanished after several weeks of stress application [16]. The discrepancy to previous studies was explained by a diurnal variation of CMS effects that were found to be more robust when the sucrose test was performed during the dark period [31]. In our study in mice, we followed the suggestion of measuring the intake behaviour during the first hours of the dark period, therefore we should have picked the most sensitive time period for measuring a CMS effect on saccharin intake. In a study of Pothion and colleagues [21] the difference between control and CMS groups of mice in a 24 hrs sucrose test was also only observed for the first 4 weeks after onset of stress. We observed strong fluctuations in the consummatory data in our experiments. These might be a consequence of (1) the short measurement interval and the consequential low intake during that time, (2) the assessment of consummatory behaviour more than once a week and (3) a possibly disturbing effect on the intake of control animals since CMS mice most of the time were kept and stressed in the same room [17].

One aim of the study was to identify potential differences on the impact of specific stressors contained in the CMS procedure on saccharin consumption and therefore, the protocol followed a fixed weekly schedule rather than being designed entirely in an unpredictable way. The preceding stressor with the largest effect on saccharin intake in the experiments was the wet cage. Our interpretation was that under this condition it was hard for the animals to rest. Indeed, keeping rats in a wet cage was used to establish an animal model of fatigue [34]. Thus, we followed the idea of a potential impact of mild disturbance of activity/sleep rhythm on saccharin consumption. Overnight illumination was applied as the sole stressor and measuring the subsequent saccharin intake was paralleled by recording homecage activity. As shown in Figure 6A, the volume of saccharin consumption during the first 2 hours at the beginning of the dark period of stressed animals dropped to a level similar to basal water consumption. In parallel, a reduction of activity was observed that was most prominent during the time when saccharin was presented. This suggests that the reduced saccharin intake was not only due to a shift of general consummatory behaviour but combined with a parallel shift and reduction in activity as observed during the same time interval. There was a moderate positive correlation of activity and intake during the time interval of saccharin presentation for LS as well as control animals. Therefore, the levels of activity and of saccharin intake are closely interwoven and the potential influence of a stressor preceding the saccharin measurement on activity changes should be considered.

In the rat version of the CMS model, changes in diurnal rhythms [35] and sleep architecture [36], [37] were reported. Further, D'Aquila et al. [31] showed the diurnal variation of CMS-induced anhedonic behaviour with its presence mainly during the active phase (dark period) of the animals. Papp and colleagues [38] concluded that the procedure causes a generalised disorganisation of internal rhythms which are postulated to play an important role in the pathophysiology of depression [39]. In mice, strain-dependent differences in locomotor activity rhythm and its changes due to daylight reversal [40], as well as a relation of sleep changes due to mild stressors - like environmental novelty - with trait anxiety [41] have been described.

Taken together, in addition to strain-dependent intake of sweetened liquids [21], [42], [43] and stress effects on the reward system [42], [44], the sensitivity to changes in activity/sleep due to the CMS procedure contributes to the final decrease in consumption behaviour. This could be of specific relevance for those studies that apply mild stressors and determine intake or preference of sweetened solutions following different stressors [21]. A consequence could be a higher variation in the anhedonic profile as observed in our study.

Besides the measurement of saccharin intake our second focus was to address additional indicators of anxiety- and depressive-like behaviour. Therefore, animals of three strains of mice were additionally characterised regarding CMS effects on anxiety-related behaviour. Furthermore, the animals were investigated in the FST, a test often used to evaluate antidepressant-like properties of substances [45]. First, BL/6J and D2Ola mice were tested in above mentioned paradigms after CMS experience of more than 10 weeks. Second, a different batch of BL/6J mice was investigated after 2 weeks of CMS experience when the decrease in saccharin intake became apparent. Third, experiments with CMS-experienced BL/6N mice also addressed the influence of illumination during behavioural testing [18]. Testing BL/6J and D2Ola mice after long-term CMS experience revealed that BL/6J mice remained unaffected by CMS, whereas D2Ola mice of the CMS group showed a decrease in anxiety-related behaviour. In the second group of BL/6J mice, after 2 weeks of CMS, again no difference in anxiety-like behaviour was seen compared to the control group. The anxiolytic-like effect of CMS in D2Ola mice is commonly appraised ‘anomalous’, yet has also been found in other chronic stress paradigms involving rats, other mouse strains and different anxiety tests [23], [46]–[52]. Generally, these findings are interpreted as either being due to blunted emotionality [52] or caused by methodological differences [48] of CMS procedures that yield a ‘classic’ anxiogenic stress response. Nevertheless, the resilience of BL/6J mice against disturbing CMS effects on anxiety-related behaviour as shown by Mineur et al. [24] could be confirmed in our study.

According to Strekalova et al. [18] bright and even moderate illumination conditions (>5 lx) can confound anxiety- and depression-related behavioural readout in chronically stressed BL/6N mice in common test paradigms by eliciting hyperlocomotion. Furthermore, chronic stress in general is supposed to exert stimulating effects on locomotor activity [53] that might mask other stress effects in anxiety- as well as in depression-related test paradigms [18]. For this reason, in our study involving BL/6N mice, illumination levels of the test apparatus were attenuated and testing was performed at the beginning of the dark phase. Under these conditions, no CMS-induced changes were observed in the DaLi. In the mHb test a CMS-induced hyperlocomotion and increased vertical exploration was paralleled by an increased time spent on the board, thus fitting into the picture drawn by Strekalova et al [18]. Since hyperlocomotion still appeared under red light conditions in the open field test in BL/6N mice (data not shown), our CMS protocol seems to exert additional effects on reactivity to a test situation that lie beyond an increased sensitivity to even moderate illumination conditions. Possibly in line with this concept are the results of all conducted FSTs in our study where no significant CMS-induced change in immobility was observed. According to the outlined concept, an increased immobility might have been concealed by CMS-induced hyperarousal, rather independent of test time and illumination.

Taken together, our findings on anxiety- and depression-related behaviour point towards a higher reactivity to a novel situation in response to CMS. This might imply the need to put the ‘anomalous’ behavioural profile in response to our CMS protocol in perspective - although this conclusion is highly speculative in respect to our limited data. Taking into account the above described inefficiency of CMS to alter anxiety-related behaviour in BL/6J mice, the presented data suggest that the occurrence of anhedonic behaviour and changes in behaviour assessed in tests for anxiety- or depression-related behaviour were uncoupled from each other.

Strain effects of CMS are further diversified by different responses of body weight gain. Whereas BL/6J and D2Ola mice showed a reduced body weight gain due to CMS, BL/6N remained entirely uninfluenced by CMS in this parameter. Apparently stressor intensity was high enough to yield an attenuation of body weight gain in D2Ola and BL/6J mice, while BL/6N mice seem to require stronger stressor application to show the same effect [18], [25]. Particularly in the face of contradictory findings reported in other CMS studies [20], [21] we consider the observed changes in body weight gain as a verification of CMS effectiveness in general rather than associating them with the phenomenology of major depression.

On the basis of the results obtained so far, our next steps in the direction of a CMS model in mice will include some modifications of the CMS procedure and the experimental design. Assessment of consummatory behaviour will be accompanied by detailed determination of homecage activity during the saccharin tests, and finally, the effects of antidepressant treatment on the diverse behavioural endpoints included in the present study will decide upon the applicability of our CMS model in general and additionally might reveal a possible relation of ‘anomalous’ behavioural changes to symptoms observed in human depression.

## Supporting Information

Figure S1 Effects of short-term CMS on saccharin consumption per 2 hrs in other mouse strains Effects of CMS on saccharin intake over a period of 3-4 weeks in Balb/c, D2JIco, FVB and NMRI mice (measurement 2x/week). White circles: control group, black circles: CMS group. First 4 data points of each graph represent basal consumption. Data represent mean±SEM, n = 8-24/group. * p<0.05, ** p<0.01 pairwise between-group comparisons (Student's t-test).
(0.45 MB TIF)

Table S1 Inter-strain comparison of saccharin intake before and after CMS application in stressed animals. Averaged saccharin intake during 2 weeks under basal conditions, the same parameter corrected for body weight and averaged intake during the first 3-4 weeks of CMS (as percentage of basal intake). a-f indicate significant differences to Balb/c, BL/6J, D2JIco, D2Ola, FVB and NMRI mice, respectively (Tukey test). Data represent mean±SEM of CMS animals only, n = 8-24/group.
(0.01 MB RTF)

## References

[1] American Psychiatric Association (1994). Diagnostic and Statistical Manual of Mental Disorder (DSM-IV).

[2] Kendler KS, Karkowski LM, Prescott CA (1999). Causal relationship between stressful life events and the onset of major depression.. Am J Psychiatry.

[3] Kessler RC (1997). The effects of stressful life events on depression.. Annu Rev Psychol.

[4] Willner P, Towell A, Sampson D, Sophokleous S, Muscat R (1987). Reduction of sucrose preference by chronic unpredictable mild stress, and its restoration by a tricyclic antidepressant.. Psychopharmacology (Berl).

[5] Anisman H, Matheson K (2005). Stress, depression, and anhedonia: caveats concerning animal models.. Neurosci Biobehav Rev.

[6] Henn FA, Vollmayr B (2005). Stress models of depression: forming genetically vulnerable strains.. Neurosci Biobehav Rev.

[7] Fuchs E (2005). Social stress in tree shrews as an animal model of depression: an example of a behavioral model of a CNS disorder.. CNS Spectr.

[8] Schmidt MV, Sterlemann V, Ganea K, Liebl C, Alam S (2007). Persistent neuroendocrine and behavioral effects of a novel, etiologically relevant mouse paradigm for chronic social stress during adolescence.. Psychoneuroendocrinology.

[9] Anisman H, Merali Z, Hayley S (2008). Neurotransmitter, peptide and cytokine processes in relation to depressive disorder: comorbidity between depression and neurodegenerative disorders.. Prog Neurobiol.

[10] de Kloet ER, Joels M, Holsboer F (2005). Stress and the brain: from adaptation to disease.. Nat Rev Neurosci.

[11] Holsboer F (2000). The corticosteroid receptor hypothesis of depression.. Neuropsychopharmacology.

[12] Linthorst AC, Reul JM (2008). Stress and the brain: solving the puzzle using microdialysis.. Pharmacol Biochem Behav.

[13] McEwen BS (2005). Glucocorticoids, depression, and mood disorders: structural remodeling in the brain.. Metabolism.

[14] Nemeroff CB (1988). The role of corticotropin-releasing factor in the pathogenesis of major depression.. Pharmacopsychiatry.

[15] Sapolsky RM (2003). Stress and plasticity in the limbic system.. Neurochem Res.

[16] Willner P (1997). Validity, reliability and utility of the chronic mild stress model of depression: a 10-year review and evaluation.. Psychopharmacology (Berl).

[17] Willner P (2005). Chronic mild stress (CMS) revisited: consistency and behavioural-neurobiological concordance in the effects of CMS.. Neuropsychobiology.

[18] Strekalova T, Spanagel R, Dolgov O, Bartsch D (2005). Stress-induced hyperlocomotion as a confounding factor in anxiety and depression models in mice.. Behav Pharmacol.

[19] Griffiths J, Shanks N, Anisman H (1992). Strain-specific alterations in consumption of a palatable diet following repeated stressor exposure.. Pharmacol Biochem Behav.

[20] Ibarguen-Vargas Y, Surget A (2008). Multifaceted strain-specific effects in a mouse model of depression and of antidepressant reversal.. Psychoneuroendocrinology.

[21] Pothion S, Bizot JC, Trovero F, Belzung C (2004). Strain differences in sucrose preference and in the consequences of unpredictable chronic mild stress.. Behav Brain Res.

[22] Ducottet C, Aubert A, Belzung C (2004). Susceptibility to subchronic unpredictable stress is related to individual reactivity to threat stimuli in mice.. Behav Brain Res.

[23] Ducottet C, Belzung C (2004). Behaviour in the elevated plus-maze predicts coping after subchronic mild stress in mice.. Physiol Behav.

[24] Mineur YS, Belzung C, Crusio WE (2006). Effects of unpredictable chronic mild stress on anxiety and depression-like behavior in mice.. Behav Brain Res.

[25] Strekalova T, Spanagel R, Bartsch D, Henn FA, Gass P (2004). Stress-induced anhedonia in mice is associated with deficits in forced swimming and exploration.. Neuropsychopharmacology.

[26] Griebel G, Stemmelin J, Scatton B (2005). Effects of the cannabinoid CB1 receptor antagonist rimonabant in models of emotional reactivity in rodents.. Biol Psychiatry.

[27] Elizalde N, Gil-Bea FJ, Ramirez MJ, Aisa B, Lasheras B (2008). Long-lasting behavioral effects and recognition memory deficit induced by chronic mild stress in mice: effect of antidepressant treatment.. Psychopharmacology (Berl).

[28] Griebel G, Simiand J, Serradeil-Le GC, Wagnon J, Pascal M (2002). Anxiolytic- and antidepressant-like effects of the non-peptide vasopressin V1b receptor antagonist, SSR149415, suggest an innovative approach for the treatment of stress-related disorders.. Proc Natl Acad Sci U S A.

[29] Li S, Wang C, Wang W, Dong H, Hou P (2008). Chronic mild stress impairs cognition in mice: from brain homeostasis to behavior.. Life Sci.

[30] Harkin A, Houlihan DD, Kelly JP (2002). Reduction in preference for saccharin by repeated unpredictable stress in mice and its prevention by imipramine.. J Psychopharmacol.

[31] D'Aquila PS, Newton J, Willner P (1997). Diurnal variation in the effect of chronic mild stress on sucrose intake and preference.. Physiol Behav.

[32] Ohl F, Holsboer F, Landgraf R (2001). The modified hole board as a differential screen for behavior in rodents.. Behav Res Methods Instrum Comput.

[33] Thoeringer CK, Sillaber I, Roedel A, Erhardt A, Mueller MB (2007). The temporal dynamics of intrahippocampal corticosterone in response to stress-related stimuli with different emotional and physical load: an in vivo microdialysis study in C57BL/6 and DBA/2 inbred mice.. Psychoneuroendocrinology.

[34] Tanaka M, Nakamura F, Mizokawa S, Matsumura A, Nozaki S (2003). Establishment and assessment of a rat model of fatigue.. Neurosci Lett.

[35] Gorka Z, Moryl E, Papp M (1996). Effect of chronic mild stress on circadian rhythms in the locomotor activity in rats.. Pharmacol Biochem Behav.

[36] Moreau JL, Scherschlicht R, Jenck F, Martin JR (1995). Chronic mild stress-induced anhedonia model of depression; sleep abnormalities and curative effects of electroshock treatment.. Behav Pharmacol.

[37] Cheeta S, Ruigt G, van PJ, Willner P (1997). Changes in sleep architecture following chronic mild stress.. Biol Psychiatry.

[38] Papp M, Gruca P, Boyer PA, Mocaer E (2003). Effect of agomelatine in the chronic mild stress model of depression in the rat.. Neuropsychopharmacology.

[39] Wehr TA, Wirz-Justice A (1982). Circadian rhythm mechanisms in affective illness and in antidepressant drug action.. Pharmacopsychiatria.

[40] Kopp C (2001). Locomotor activity rhythm in inbred strains of mice: implications for behavioural studies.. Behav Brain Res.

[41] Tang X, Xiao J, Parris BS, Fang J, Sanford LD (2005). Differential effects of two types of environmental novelty on activity and sleep in BALB/cJ and C57BL/6J mice.. Physiol Behav.

[42] Cabib S, Puglisi-Allegra S (1996). Stress, depression and the mesolimbic dopamine system.. Psychopharmacology (Berl).

[43] Bachmanov AA, Tordoff MG, Beauchamp GK (2001). Sweetener preference of C57BL/6ByJ and 129P3/J mice.. Chem Senses.

[44] Cabib S, Puglisi-Allegra S (1996). Different effects of repeated stressful experiences on mesocortical and mesolimbic dopamine metabolism.. Neuroscience.

[45] Cryan JF, Mombereau C (2004). In search of a depressed mouse: utility of models for studying depression-related behavior in genetically modified mice.. Mol Psychiatry.

[46] D'Aquila PS, Brain P, Willner P (1994). Effects of chronic mild stress on performance in behavioural tests relevant to anxiety and depression.. Physiol Behav.

[47] Gronli J, Murison R, Fiske E, Bjorvatn B, Sorensen E (2005). Effects of chronic mild stress on sexual behavior, locomotor activity and consumption of sucrose and saccharine solutions.. Physiol Behav.

[48] Ducottet C, Griebel G, Belzung C (2003). Effects of the selective nonpeptide corticotropin-releasing factor receptor 1 antagonist antalarmin in the chronic mild stress model of depression in mice.. Prog Neuropsychopharmacol Biol Psychiatry.

[49] Rossler AS, Joubert C, Chapouthier G (2000). Chronic mild stress alleviates anxious behaviour in female mice in two situations.. Behav Processes.

[50] Li S, Wang C, Wang M, Li W, Matsumoto K (2007). Antidepressant like effects of piperine in chronic mild stress treated mice and its possible mechanisms.. Life Sci.

[51] Kopp C, Vogel E, Rettori MC, Delagrange P, Misslin R (1999). The effects of melatonin on the behavioural disturbances induced by chronic mild stress in C3H/He mice.. Behav Pharmacol.

[52] Kompagne H, Bardos G, Szenasi G, Gacsalyi I, Harsing LG (2008). Chronic mild stress generates clear depressive but ambiguous anxiety-like behaviour in rats.. Behav Brain Res.

[53] Cancela LM, Bregonzio C, Molina VA (1995). Anxiolytic-like effect induced by chronic stress is reversed by naloxone pretreatment.. Brain Res Bull.

