# Supplementary figures and images for: Chronic Mild Stress (CMS) in Mice: Of Anhedonia, ‘Anomalous Anxiolysis’ and Activity
Source: PLoS One. 2009 Jan 29;4(1):e4326. doi: 10.1371/journal.pone.0004326 (PMC2627902; doi:10.1371/journal.pone.0004326)

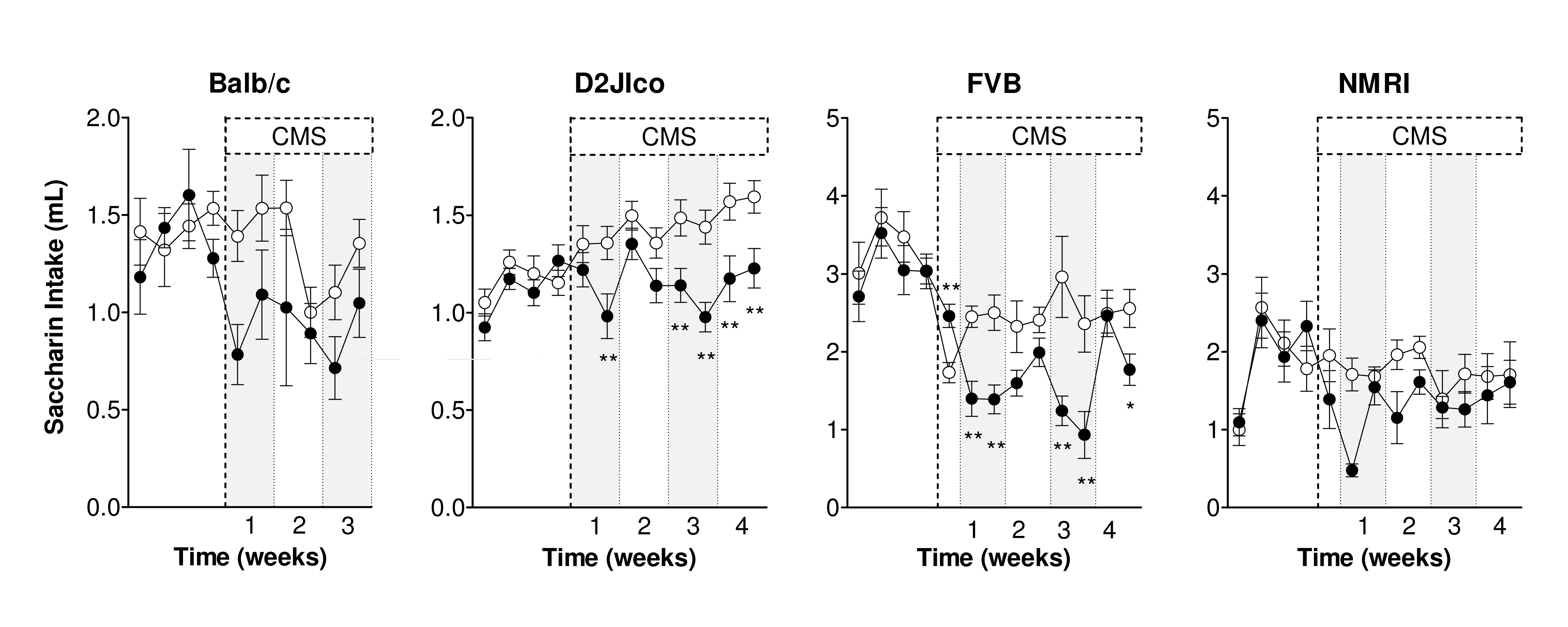

Supplement: Figure S1 — Effects of short-term CMS on saccharin consumption per 2 hrs in other mouse strains Effects of CMS on saccharin intake over a period of 3-4 weeks in Balb/c, D2JIco, FVB and NMRI mice (measurement 2x/week). White circles: control group, black circles: CMS group. First 4 data points of each graph represent basal consumption. Data represent mean±SEM, n = 8-24/group. * p<0.05, ** p<0.01 pairwise between-group comparisons (Student's t-test). (0.45 MB TIF) [file pone.0004326.s001.tif]
