# Supplementary material for: Chronic Mild Stress (CMS) in Mice: Of Anhedonia, ‘Anomalous Anxiolysis’ and Activity
Source: PLoS One. 2009 Jan 29;4(1):e4326. doi: 10.1371/journal.pone.0004326 (PMC2627902; doi:10.1371/journal.pone.0004326)
Supplement: Table S1 — Inter-strain comparison of saccharin intake before and after CMS application in stressed animals. Averaged saccharin intake during 2 weeks under basal conditions, the same parameter corrected for body weight and averaged intake during the first 3-4 weeks of CMS (as percentage of basal intake). a-f indicate significant differences to Balb/c, BL/6J, D2JIco, D2Ola, FVB and NMRI mice, respectively (Tukey test). Data represent mean±SEM of CMS animals only, n = 8-24/group. (0.01 MB RTF) [file pone.0004326.s002.rtf]

	Basal Intake (mL)	Basal Intake / Body Weight (mL/g)	CMS Intake (% Basal)	
Balb/c	1,37 ± 0,09 (b,e,f)	0,05 ± 0,003 (b,e)	70,7 ± 11,6 (c)	
BL/6J	2,12 ± 0,1 (a,c,d,e)	0,071 ± 0,003 (a,c,d,e,f)	65,6 ± 3,2 (c)	
D2JIco	1,12 ± 0,04 (b,e,f)	0,044 ± 0,002 (b,e)	105 ± 5,2 (a,b,d,e,f)	
D2Ola	1,44 ± 0,05 (b,e,f)	0,051 ± 0,003 (b,e)	69,8 ± 3,3 (c)	
FVB	3,08 ± 0,15 (a,b,c,d,f)	0,092 ± 0,005 (a,b,c,d,f)	55 ± 3,3 (c)	
NMRI	1,95 ± 0,22 (a,c,d,e)	0,04 ± 0,003 (b,e)	71,4 ± 6,4 (c)	
